# Multifunctional LiClO4 for Polymerization Regulation, Ionic Conduction, and Interphase Formation in In Situ Poly(tetrahydrofuran) Gel Polymer Electrolytes

**DOI:** 10.3390/polym18192379

**Published:** 2026-09-29

**Authors:** Si-Han Peng, Wei-Fan Kuan, Shingjiang Jessie Lue

**Affiliations:** 1 Department of Chemical and Materials Engineering, Chang Gung University, Guishan District, Taoyuan City 333, Taiwan; 2 Center for Sustainability and Energy Technologies, Department of Chemical and Materials Engineering, Chang Gung University, Guishan District, Taoyuan City 333, Taiwan; 3 Department of Safety, Health and Environmental Engineering, Ming-Chi University of Technology, Taishan District, New Taipei City 243, Taiwan

**Keywords:** gel polymer electrolyte, in situ polymerization, poly(tetrahydrofuran), lithium perchlorate, lithium perchlorate-assisted formation

## Abstract

In situ polymerized gel polymer electrolytes (GPEs) offer a promising route to improve electrode–electrolyte interfacial compatibility in quasi-solid-state lithium metal batteries. Although LiClO_4_ has been employed in such systems, its role beyond a lithium-ion source has not been systematically investigated. Here, we report a two-component electrolyte consisting of 1 M LiClO_4_ and 0.3 M BF_3_-THF (1-0.3PTHF) that enables the in situ formation of poly(tetrahydrofuran) (PTHF) with substantially reduced initiator loading. The incorporation of LiClO_4_ not only provides mobile Li^+^ ions but also facilitates polymerization. Possible ionic interactions between LiClO_4_ species and growing polymer chains may contribute to the improvement. The 1-0.3PTHF exhibited a narrow mass distribution (Đ = 1.23) and a low degree of crystallinity (4.7%) compared to commercial PTHF (Đ = 3.25) and the PTHF polymerized without LiClO_4_. The in situ 1-0.3PTHF delivered a bulk ionic conductivity of 2.75 × 10^−3^ S cm^−1^, an electrochemical window of 5.06 V, and stable lithium plating/stripping up to 3 mA cm^−2^, suggesting its promise as a GPE. Post-mortem XPS confirms the coexistence of B-containing species and LiCl within the SEI. Full cell tests demonstrated the preliminary feasibility of the 1-0.3PTHF GPE with LFP, LCO, and NCM cathodes. This work highlights the multifunctional role of LiClO_4_ in in situ polymerized PTHF. The resulting 1-0.3PTHF electrolyte simultaneously regulates polymerization, enhances ionic conduction, and promotes protective SEI formation. These findings broaden the role of lithium salts beyond lithium-ion sources to polymerization modulators in in situ polymerized GPEs.

## 1. Introduction

The growing demand for high-performance energy storage devices drives innovation in materials to improve efficiency, stability, and energy density [1,2]. Notably, using Li anode and solid-state electrolyte (SSE) enhances both gravimetric and volumetric energy density relative to the State-of-the-Art Li-ion batteries (LIBs), making solid-state Li metal battery (SSLMB) systems highly attractive to researchers [3]. The employment of SSEs is promising to address almost all forms of interfacial instability, such as roughness control of Li deposits, associated with premature failure and safety hazards from instability of liquid electrolytes (LEs) toward Li metallic anodes [4,5,6]. However, the intrinsic electrode contact of freestanding SSEs via ex situ processes remains challenging, as the resulting large interfacial impedance severely limits overall battery performance [7,8,9]. A variety of interfacial engineering strategies have been implemented to improve surface contact, including modulation of surface energy [10,11], quasi-type gel polymer electrolytes (GPEs) [12,13,14], and employment of stacking pressure [15,16]. Nonetheless, these methods are often cost-intensive, power-demanding and time-consuming, casting doubts on their compatibility with large-scale production and diverse battery configurations [17].

In situ polymerization presents a compelling approach for developing next-generation SSEs with excellent interfacial contact toward electrodes [9,18,19,20,21]. In situ polymerization transforms a monomer-based liquid precursor into a quasi-solid-state GPE inside the battery under specific initiating conditions. The fluidity of liquid precursor ensures uniform electrode wettability and penetration into the intrinsic voids of porous electrodes, followed by in situ polymerization to form a GPE directly inside the battery [22]. Encouragingly, the in situ synthesis of GPE is compatible with the current preparation process of LMBs employing LE, making it more conducive to mass production for energy storage devices, in comparison with ex situ SSE manufacturing processes [18,23]. It mitigates the drawbacks of LEs and SSEs while integrating their advantages, presenting a promising electrolyte design for SSLMBs [9]. Yet, careful initiator selection is crucial, as it governs gel integrity and solid electrolyte interphase (SEI) stability, directly impacting battery performance. Azobisisobutyronitrile (AIBN), which activates free-radical reaction under thermal condition (60 °C), has been popularly used as an initiator due to its advantages of having no by-product formation and ease of control [24,25,26]; however, this approach raises concerns about side effects due to the presence of free radicals [27], and has been reported to exhibit poor compatibility with Li metal anodes [9]. To address such interfacial instability, multifunctional initiators have been developed to construct robust GPE systems with stable SEIs [28,29,30,31]. Recently, we employed boron trifluoride tetrahydrofuran (BF_3_-THF) as a novel initiator for in situ preparation of poly(tetrahydrofuran) (PTHF) [32]. This oxonium ion-like initiator directly converted monomer into PTHF without generating intermediates and introducing impurities, and contributed to the formation of B and F-rich SEI to afford robust protection of Li metal anode.

In addition, electrolyte-salt-induced cationic ring-opening polymerization, where electrolyte salts work as both Li^+^-conducting salt and initiator simultaneously, has attracted much attention as a facile in situ gelation strategy to avoid the introduction of impurities [28]. Guo et al. readily converted a commercialized LE (1 M LiTFSI in a 1,3-dioxolane and 1,2-dimethoxyethane (DOL/DME) with a mass ratio of 1:1) to a solid–liquid hybrid electrolyte by using 2 M LiPF_6_ to initiate polymerization of 1,3-dioxolane (DOL) [33]. Wu et al. reported a novel eutectic solution of 1,3,5-trioxane (TXE) and succinonitrile that was converted into a GPE through in situ polymerization initiated by lithium difluoro(oxalato)borate (LiDFOB) salt, where all experiments were conducted at an elevated 80 °C [34]. Wang et al. reported an initiator-free in situ 3.5 M LiFSI-DOL GPE that integrated a high ionic conductivity of 7.9 mS cm^−1^ along with a high Li^+^ transference number of 0.82 [22]. The superb advantages of using Li-conducting salt as an initiator for the in situ polymerization process include commercial raw materials and being free of extra initiators, making this a promising in situ gelation strategy. To our knowledge, these methods are inevitably accompanied with either extremely high concentration of Li salt addition or high-temperature conditions to promote an effective gelation process. Wang et al. have studied the behavior of LiTFSI-DOL electrolyte, where the concentrations of LiTFSI were less than 3 M, and found that such DOL electrolytes were not polymerized into immobile PDOL [22]. Guo et al. further proposed that a concentrated LiPF_6_ was needed to ensure proper decomposition of Li salt, where the product was used for initiation of DOL [33]. Considering that the concentrated salt systems and temperature driving conditions were not favorable for scale-up production and universal battery systems, advancing polymerization will require innovative electrolyte design.

Lithium perchlorate (LiClO_4_) has been reported to catalyze epoxide ring-opening polymerization for the preparation of cross-linked epoxide-containing polyether [35,36,37]. Moreover, multifunctional additives that participate in polymerization can simultaneously improve polymerization efficiency but also facilitate interfacial construction [38,39]. Huang et al. previously reported a LiClO_4_-containing, in situ polymerized PTHF electrolyte, in which BF_3_-etherate served as a functional initiator and contributed to the formation of LiF and B-O species in the SEI, whereas LiClO_4_ was used primarily as the Li^+^-conducting salt [31]. The present study further examines LiClO_4_ as a polymerization-assisting additive and a contributor to SEI composition beyond its conventional role as a Li^+^ source. In this work, a two-component system consisting of 1 M LiClO_4_ salt and 0.3 M BF_3_-THF initiator (1-0.3PTHF) was developed to enable in situ polymerization of THF under reduced initiator loading. Here, LiClO_4_ not only serves as a Li^+^ source but also acts as a polymerization-assisting additive during cationic ring-opening polymerization. Bare PTHF systems in the absence of LiClO_4_ were employed as the counterparts to demonstrate the contribution of LiClO_4_. The resulting 1-0.3PTHF offers several advantages such as low-cost raw materials, concentration-efficient composition, and room-temperature processing. Polymer characterization, electrochemical performance, and post-mortem analysis were systematically conducted to investigate the multifunctional role of LiClO_4_. This work broadens our understanding of the role of lithium salts beyond lithium-ion sources to polymerization modulators in in situ polymerized GPEs.

## 2. Experimental Section

**Figure 1:**
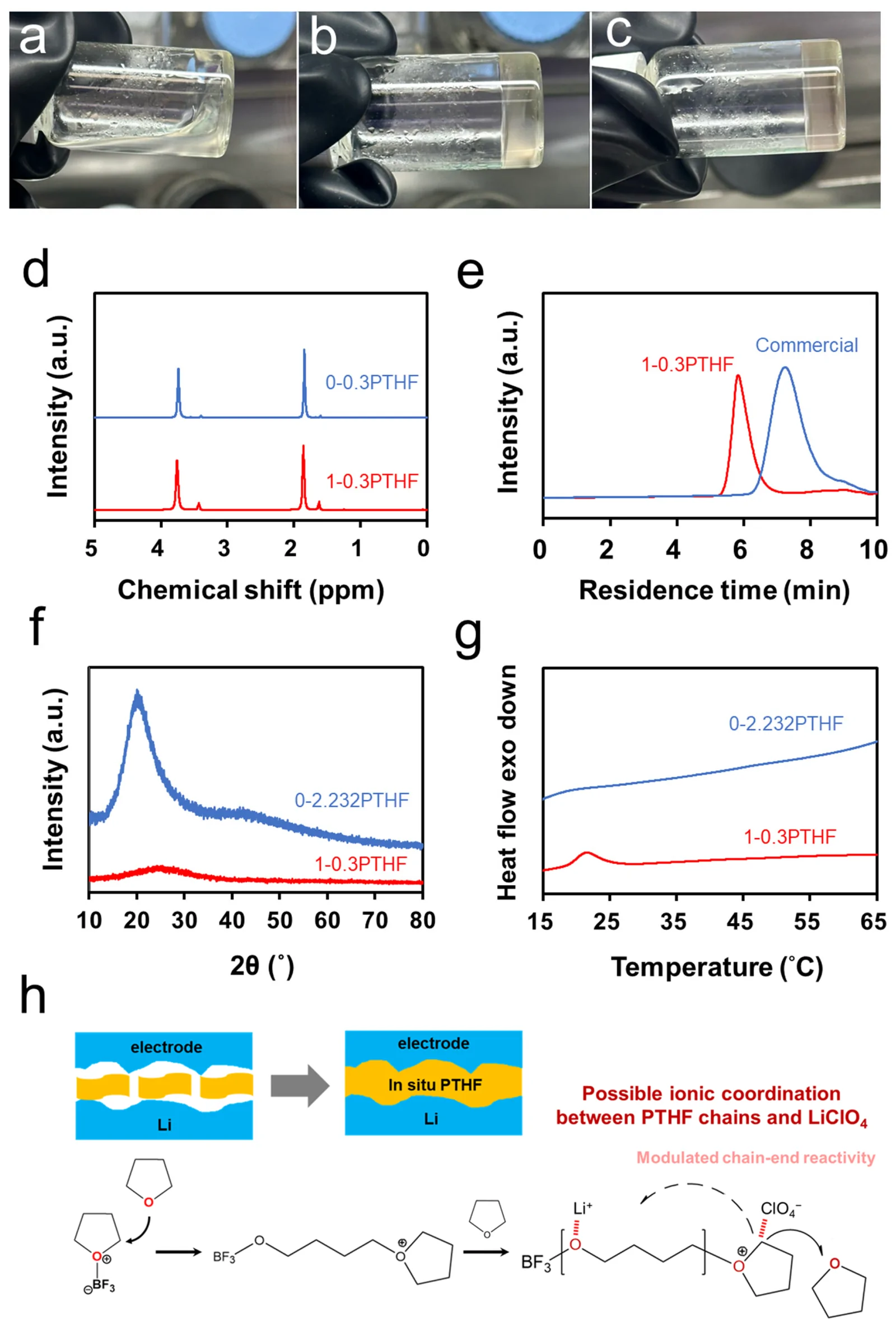
The digital images of in situ 1-0.3PTHF gel after (**a**) 5 h, (**b**) 9 h, and (**c**) overnight at room temperature; (**d**) 1H NMR spectra of bare 0-0.3PTHF and 1-0.3PTHF. (**e**) GPC profile of commercial PTHF and 1-0.3PTHF. (**f**) XRD pattern and (**g**) DSC analysis of 1-0.3PTHF gel and bare 0-2.232PTHF. (**h**) Schematic illustration of reaction route of THF polymerization and possible ionic interactions during LiClO_4_-assisted PTHF formation.

All polymerization experiments were carried out in an Ar-filled glovebox with O_2_ and H_2_O levels below 0.5 ppm. LiClO_4_ (anhydrous, ACS, 95% min, Alfa Aesar, Ward Hill, MA, USA) and BF_3_-THF (Sigma-Aldrich, St. Louis, MO, USA) solutions in THF (anhydrous, ≥99.9%, inhibitor-free, Sigma-Aldrich) were prepared separately with adjusted stoichiometries so that, upon mixing at a 1:1 (*v*/*v*) ratio, the final concentrations of LiClO_4_ and BF_3_-THF were 1 M and 0.3 M, respectively. The resulting mixture formed the 1-0.3PTHF gel. Figure 1h illustrates the proposed mechanism of THF polymerization initiated by BF_3_–THF. In situ assembly of 1-0.3PTHF was fabricated by immersing a 18 mm diameter of glass fiber (Whatman, Little Chalfont, Bucks, UK, 420 μm in thickness) in the polymeric precursor composed of 1 M LiClO_4_ + 0.3 M BF_3_-THF for 1 h to enable initial oligomer formation. Glass fiber (GF) was employed as the support for electrolyte uptake. Afterward, the impregnated GF containing PTHF oligomer (GF-supported 1-0.3PTHF) was stacked with electrode materials and sealed for cell assembly. As references, bare PTHF systems in the absence of LiClO_4_ were prepared using polymeric solutions containing BF_3_-THF at concentrations of 0.3, 0.553, 1.116, and 2.232 M in THF, denoted as 0-0.3PTHF, 0-0.553PTHF, 0-1.116PTHF, and 0-2.232PTHF, respectively. Among them, 0-2.232PTHF was prepared following the procedure reported in our previous work [32], while 0-0.3PTHF, 0-0.553PTHF, and 0-1.116PTHF were prepared analogously by adjusting the BF_3_-THF concentration accordingly. Experimental details including materials, characterization methods, and electrochemical measurements are provided in the Supplementary Materials.

## 3. Results and Discussion

### 3.1. LiClO_4_-Assisted Cationic Ring-Opening Polymerization of THF

As previously reported by our group [32], the gelation behavior of bare PTHF formulations without LiClO_4_ is strongly dependent on the BF_3_-THF initiator concentration, a Lewis acid that acts as an electrophilic agent for polymerization. Among the investigated systems, 0-2.232PTHF exhibited continuous gel evolution and uniform THF conversion, leading to successful gelation. Reducing the BF_3_-THF content to half-dosage (0-1.116PTHF) preserved the uniform transformation behavior, albeit with a noticeably prolonged gelation time. Further reduction to quarter-dosage (0-0.553PTHF) resulted in insufficient polymerization, as the material remained visibly flowable even after prolonged storage. These observations confirm that THF polymerization initiated by Lewis acids requires a high initiator concentration to achieve significant polymer formation [40,41]. On this basis, we have conducted preliminary studies of combining a Li salt with BF_3_-THF to enable PTHF formation at reduced initiator concentrations (with details shown in Figures S1 and S2). Particularly, the LiClO_4_-PTHF system produced a more homogeneous polymer network at a lithium salt concentration of 1 M. In this work, we primarily evaluate whether LiClO_4_ can act as a polymerization-assisting additive using 1-0.3PTHF as a case study.

**Figure 2:**
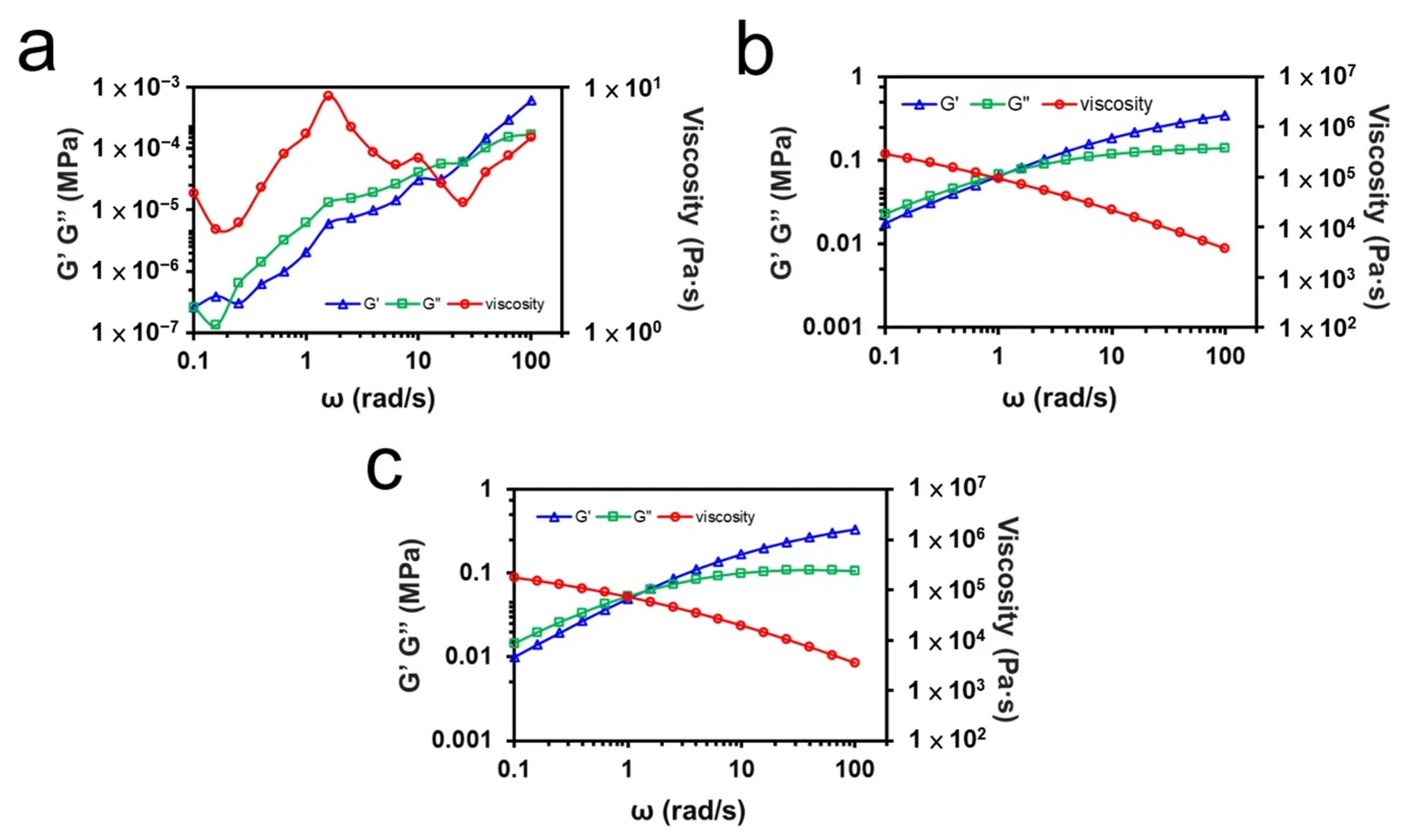
Rheological frequency sweep measurements of (**a**) 0-0.3PTHF, (**b**) 1-0.3PTHF, and (**c**) 0-2.232PTHF. The storage modulus (G′) and loss modulus (G″) were measured as a function of angular frequency (ω) to evaluate the viscoelastic behavior.

Firstly, 0-0.3PTHF was prepared as a control group where no visible change in fluidity was observed even after 48 h, as shown in Figure S3, which is associated with deficient polymerization process at a low BF_3_-THF concentration. Digital images in Figure 1a–c revealed a gradual transformation of the 1-0.3PTHF precursor, where the initially free-flowing liquid became viscous after 5 h of initiation, suggesting oligomer formation (Figure 1a). After 9 h, the 1-0.3PTHF became highly viscous (Figure 1b), and prolonged storage yielded a uniform gel with immovable characteristic (Figure 1c). Notably, transient spatial heterogeneity was observed during the early stage of gelation, with coexisting regions exhibiting different fluidities, as shown in Figure 1a. Nonetheless, a homogeneous gel was ultimately obtained without phase separation, as indicated in Figure 1c. Compared to the bare PTHF system, this discontinuous gel evolution suggests a modified initiation environment in the presence of LiClO_4_. Additionally, the elastic properties of 0-0.3PTHF and 1-0.3PTHF were investigated, as reported in Figure 2a,b. The rheological characteristics of 1-0.3PTHF showed a significant increase in both the elastic/storage modulus (G′) and the viscous/loss modulus (G″). Meanwhile, its viscosity was approximately four orders of magnitude higher than that of the 0-0.3PTHF, suggesting rising contribution of PTHF chains and development of viscoelastic gel structure. A crossover between G′ and G″ was observed at an angular frequency of 1 rad s^−1^ (the intersection of G′ and G″), after which G′ became dominant (G′ > G″) across the measured range, indicative of the transition to a solid-like material. Encouragingly, the 1-0.3PTHF formulation with a markedly reduced BF_3_-THF content showed more pronounced gel formation compared to the system without LiClO_4_ and exhibited solid-like behavior comparable to that of the highly concentrated 0-2.232BF_3_-THF formulation, as shown in Figure 2c.

The polymer characterization of 1-0.3PTHF gel was performed and compared with the reference PTHF. Unless otherwise specified, all PTHF samples were allowed to polymerize for 48 h at room temperature before polymer characterization. Additionally, ^1^H NMR analysis confirms that the product was PTHF (Figure 1d). The spectrum exhibited two characteristic peaks at 3.4 ppm and 1.6 ppm, corresponding to –CH_2_–O– and –CH_2_–CH_2_– repeating units of the PTHF backbone, respectively. In contrast, the two peaks were barely detectable in the bare 0-0.3PTHF, consistent with insufficient polymerization at the reduced BF_3_ concentration (0.3 M) in the absence of LiClO_4_. Additionally, the two broad peaks without split at 3.71 ppm and 1.81 ppm were predominantly assigned to be low-molecular-weight PTHF rather than residual liquid THF, consistent with previous reports [31,32]. Based on the integrated areas at 3.4 and 3.71 ppm, the THF conversion was estimated to increase from approximately 2% for 0-0.3PTHF to 12% for 1-0.3PTHF. The conversion may be underestimated due to contributions from low-molecular-weight PTHF at 3.71 ppm. Nonetheless, the higher conversion, together with the gelation behavior and rheological response, supports the enhanced PTHF formation in the presence of LiClO_4_. The number-average molecular weight (M_n_) of 1-0.3PTHF was estimated to be 3.46 × 10^3^ Da, higher than 2.9 × 10^3^ Da of commercial PTHF, as indicated by the GPC profile (Figure 1e). These results confirm the successful chain-growth polymerization of THF in 1-0.3PTHF gel, producing chains of similar length to the 0-2.232PTHF (3.5 × 10^3^ Da) [32]. Notably, 1-0.3PTHF displayed a sharper and more symmetric GPC curve than commercial PTHF, indicating a more uniform molecular weight distribution with a dispersity (Đ) of 1.23 compared to 3.25 for the commercial PTHF. As a reference, the reported bare 0-2.232PTHF exhibited a broader distribution (Đ of 1.88) [32].

In addition, LiClO_4_ may provide either charged clusters (contact ion pairs or aggregates) or transient Li–O coordination that disrupts local chain packing [42,43]. As shown in Figure 1f, the XRD analysis showed that both 0-2.232PTHF and 1-0.3PTHF were amorphous. In particular, 1-0.3PTHF showed a flatter XRD pattern compared with 0-2.232PTHF, indicating that PTHF segments were distorted from the sterically crowded environment and electrostatic interaction. The flatten XRD pattern is in line with the literature as increment in LiClO_4_ composition to polymer [36]. Additionally, the XRD pattern of 1-0.3PTHF suggested LiClO_4_ dissociation within polymer matrix, as evidenced from absence of LiClO_4_ crystal peaks. This finding suggested no pure form of LiClO_4_ crystal. The incorporating LiClO_4_ shifted the diffraction peak from 21° (0-2.232PTHF) to 24° in 1-0.3PTHF, in agreement with the findings of Huang et al. [31]. DSC analysis provides thermal transition study to compare 0-2.232PTHF and 1-0.3PTHF (Figure 1g). An additional melting endotherm was observed for the 1-0.3PTHF gel, whereas no such transition appeared in 0-2.232PTHF. The melting enthalpy of the 1-0.3PTHF was determined to be 0.58 kJ mol^−1^, compared to the reported heat of fusion of 12.4 kJ mol^−1^ for completely crystalline PTHF [44]. The melting enthalpy is based on the mole of repeating units. By using these values, the crystallinity of 1-0.3PTHF was estimated to be 4.7%, obtained by normalizing its melting enthalpy to that of fully crystalline PTHF. It was reported that the formation of coordinative complexes due to Li^+^-ether oxygen interactions contributed to small polymer crystalline domains [36,45], as manifested in the appearance of an endothermic peak in the DSC result. The well-dispersed LiClO_4_ in 1-0.3PTHF broadened the amorphous halo and induced a low degree of crystallinity, while the system remained predominantly amorphous, as confirmed by both XRD and DSC analyses.

Collectively, these results indicate that the presence of LiClO_4_ alters the gelation behavior of THF and facilitates PTHF formation at a substantially reduced BF_3_-THF concentration. Whereas 0-0.3PTHF remained fluid because of insufficient initiation, 1-0.3PTHF clearly exhibited solid-like characteristics at the same BF_3_-THF concentration. Chini et al. showed that LiClO_4_ can promote ring-opening reactions of oxygen-containing cyclic compounds through ionic interactions [46]. In addition, previous studies have demonstrated that ionic interactions can modulate the reactivity of charged intermediates during ring-opening polymerization [47,48,49,50,51,52]. On this basis, possible formation of coordinative complexes (Li-O coordination and ClO_4_^−^ anions with positively charged terminal) may alter the local ionic environment and thereby influence chain initiation or propagation, as schematically proposed in Figure 1h. Such interactions may account for the observed changes in PTHF gelation, molecular weight distribution, and crystallization behavior. Nevertheless, the present results do not directly identify these interactions, and direct characterization of the propagating species would be required to establish the proposed polymerization mechanism. LiClO_4_ is therefore described here as a polymerization-assisting additive rather than an independent initiator.

**Figure 3:**
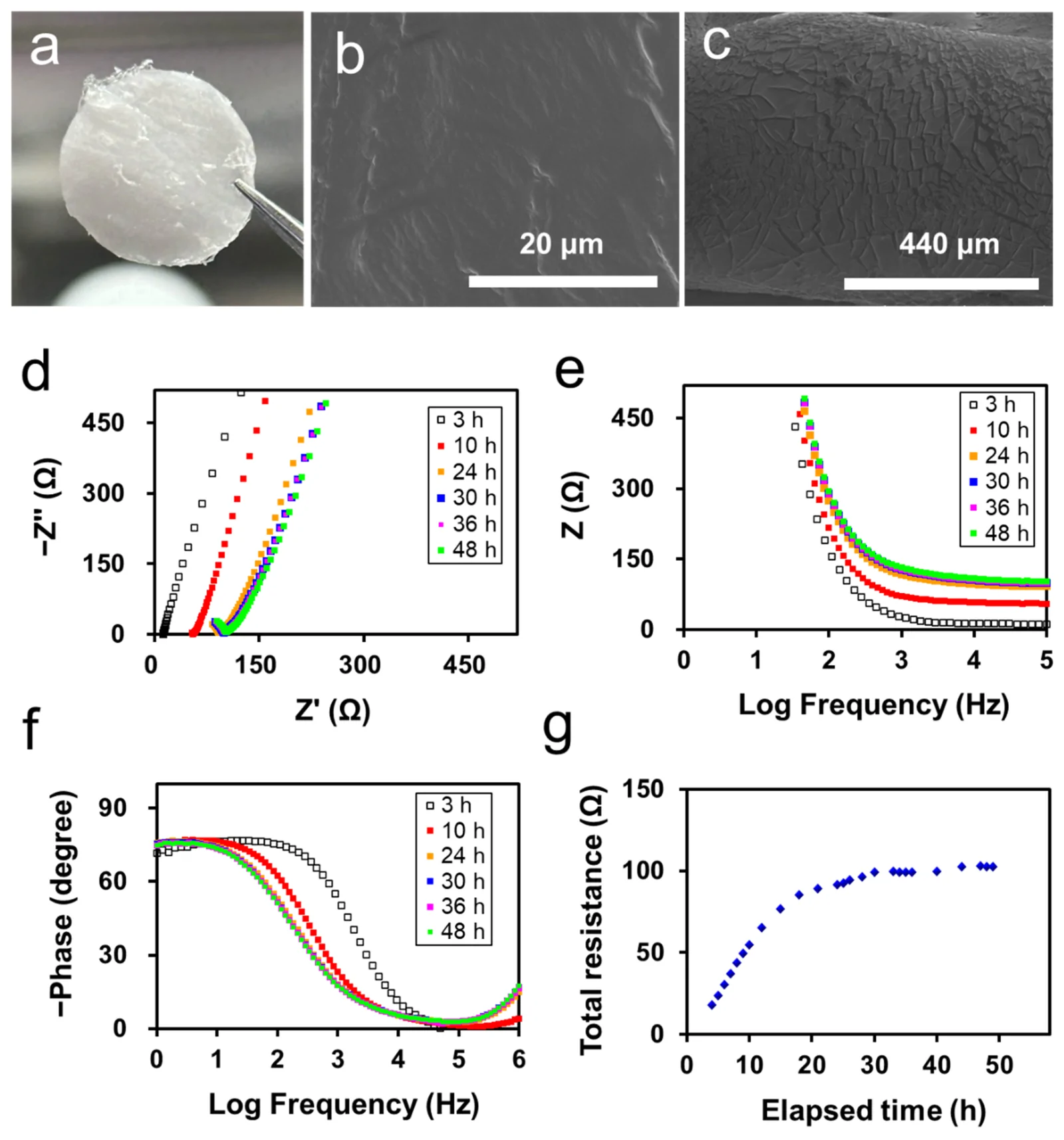
(**a**) Optical image of glass fiber (GF)-supported 1-0.3PTHF, and the corresponding (**b**) top-view and (**c**) cross-sectional SEM. (**d**) EIS spectra of stainless-steel (SS) symmetric cells using in situ 1-0.3PTHF at different elapsed times with the corresponding (**e**,**f**) Bode plots and (**g**) total resistance (R_b_ + R_ct_) of the cell at various elapsed times.

Figure 3a shows optical image of the GF-supported 1-0.3PTHF GPE, while the corresponding SEM (Figure 3b) reveals a dense morphology in contrast to the fibrous and porous texture of pristine GF (Figure S4). The cross-sectional SEM image (Figure 3c) further confirms that the PTHF gel was homogeneously incorporated into fiber networks throughout the support. This dense and flat morphology indicated that 1-0.3PTHF was uniformly bonded to the GF substrate, forming an interconnected PTHF electrolyte network. In addition, LiClO_4_ salt was completely dissolved within polymer matrix, as no salt precipitation was observed on the 1-0.3PTHF GPE surface. This observation aligns with the XRD result (Figure 1f), where no crystalline peaks corresponding to LiClO_4_ were detected.

### 3.2. LiClO_4_-Enhanced Ionic Transport and Electrochemical Performance

EIS was employed to in situ monitor the dynamic polymerization of THF, where the impedance response varied markedly as the monomer transitioned from a flowable liquid to a gel-like electrolyte at different polymerization stages [32]. The electrochemical behavior was examined using a stainless-steel symmetric cell assembled with the GF-supported 1-0.3PTHF GPE. As shown in Figure 3d, the Nyquist plots displayed a progressive shift in the x-intercept toward higher impedance up to 24 h, after which the resistance gradually stabilized. The x-intercept was used to correspond to total resistance. At 3 h, the precursor remained largely liquid, enabling excellent electrode wettability and yielding the lowest total resistance dominated by bulk resistance (R_b_), as evidenced by the impedance plateau in the Bode plot (Figure 3e). As polymerization proceeded (10–24 h), the increasing total resistance reflected the conversion of THF to PTHF, where interfacial resistance (R_ct_) became the primary contributor. The corresponding plateau in the Bode plot shifted to higher impedance (Figure 3e), while the phase angle (Figure 3f) evolved from a steep to a gentler slope, indicating the emergence of R_ct_. Between 30 and 48 h, overlapping Nyquist plots with similar total resistance possibly suggested equilibrium polymerization. The time-dependent total resistance (Figure 3g) inferred that the major transformation occurred within the first 24 h, accompanied by a sharp rise in impedance, followed by the reaction of unpolymerized THF, as reflected by a flatter gradient. The impedance eventually stabilized, signifying the formation of a stable 1-0.3PTHF GPE. Despite the reduced BF_3_-THF concentration (0.3 M), the stabilization period was comparable to that of bare 0-2.232PTHF [32], supporting the role of LiClO_4_ as a polymerization-assisting additive that compensates for the reduced initiator content.

**Figure 4:**
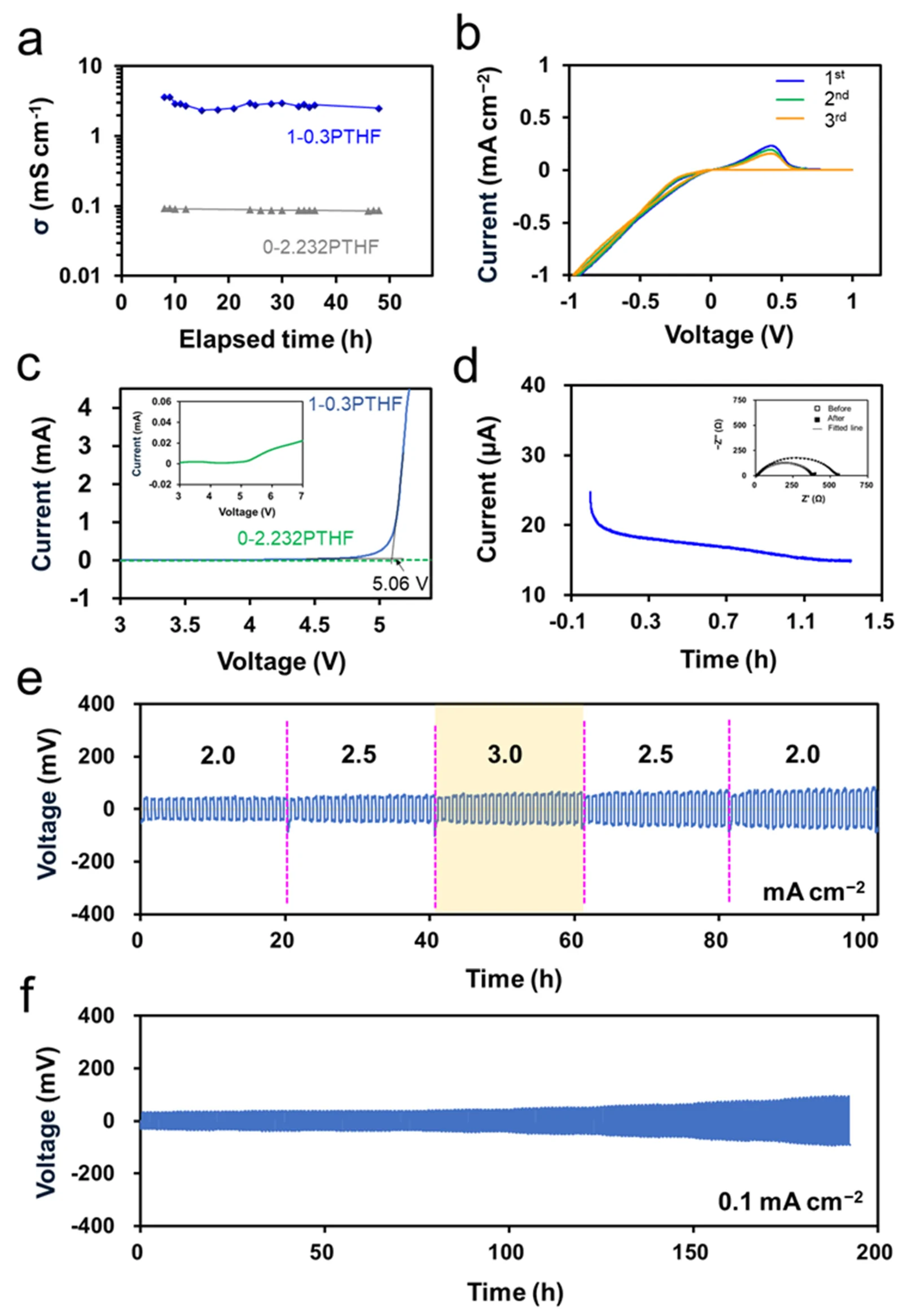
(**a**) Comparison of bulk ionic conductivity for the Li symmetric cells using in situ 1-0.3PTHF and bare 0-2.232PTHF at various elapsed times, (**b**) CV of Li–SS cell using in situ 1-0.3PTHF at a sweep rate of 1 mV s^−1^ (normalized to the SS electrode area), (**c**) comparison of ESW for in situ 1-0.3PTHF and bare 0-2.232PTHF, (**d**) chronoamperometry profile of Li symmetric cell using in situ 1-0.3PTHF at 10 mV (inset: Nyquist plots before and after the polarization test), (**e**) GCD profile of Li symmetric cell employing in situ 1-0.3PTHF under stepwise current densities from 2 to 3 mA cm^−2^, and switched back to 2 mA cm^−2^. (**f**) GCD profile at 0.1 mA cm^−2^ after high-current-density cycles.

Next, EIS measurements were conducted on a Li symmetrical cell with 1-0.3PTHF GPE to in situ monitor the change in bulk ionic conductivity versus time. The conductivity was calculated from the fitted R_b_ using an equivalent circuit model (Figure S5) and compared with that of the bare 0-2.232PTHF. As shown in Figure 4a, the ionic conductivity of 1-0.3PTHF was initially higher (approximately 3.65 × 10^−3^ S cm^−1^), followed by a mild decrease, and approached a constant value at an average of 2.75 × 10^−3^ S cm^−1^. The stabilization of R_b_ corresponded well with the gelation time identified in Figure 1a–c, indicating a strong correlation between bulk transformation and ionic transport. The long-time ionic conductivity suggested that R_b_ remained nearly constant over longer duration, supporting that the impedance evolution observed between 10 and 48 h (Figure 3d–g) was governed by R_ct_ rather than R_b_. Notably, the ionic conductivity of 1-0.3PTHF was about one order of magnitude higher compared to the bare 0-2.232PTHF. Such enhancement is primarily attributed to the presence of LiClO_4_ salt, which increases the number of charge carriers (free lithium cations) and promotes Li transport through transient coordination with ether oxygen atoms. It should therefore be distinguished from the polymerization-assisting effect of LiClO_4_. Accordingly, LiClO_4_ serves a dual function, acting as both Li^+^ source and a facilitator of in situ polymer formation.

Figure 4b shows the Li stripping/plating profile of the in situ formed 1-0.3PTHF, where a strong peak at 0.43 V corresponding to Li stripping on the SS electrode during the anodic scan was observed at room temperature. The current response for the 1-0.3PTHF was significantly enhanced compared with that of the bare 0-2.232PTHF [32], as evidenced by the sloped cathodic curve and the increase in anodic peak current from approximately 10 to 215 mA cm^−2^, which reflects the facilitation of Li stripping/plating reactions by the incorporated LiClO_4_ salt. ESW was further examined to assess oxidative stability of the 1-0.3PTHF compared with the bare 0-2.232PTHF (Figure 4c). The oxidative stability onset was determined from the potential-axis intercept of the linear fit to the rising oxidation current. The reduced BF_3_-THF concentration may weaken BF_3_-polymer coordination [32] and was therefore expected to compromise voltage tolerance (5.2 V for 0-2.232PTHF). The 1-0.3PTHF still exhibited an oxidation onset of 5.06 V. This behavior can be associated with the formation of coordinative complexes between cations, anions, and polymer chains. Such coordination bonds, evidenced by local ordering of polymer chains and the linear T_g_ increase with LiClO_4_ content reported previously [36,52], are proposed to partially compensate for the loss of voltage tolerance. These findings highlight a trade-off between ionic transport and electrochemical stability in the 1-0.3PTHF.

The Li^+^-ion transport capability for the 1-0.3PTHF was evaluated by determination of t_Li+_. It is important to note that the Li/1-0.3PTHF/Li symmetric cell was preconditioned by galvanostatic cycling at 0.1 mA cm^−2^ for 30 min per cycle over four cycles prior to the chronoamperometry measurement. The polarization curve of 1-0.3PTHF is shown in Figure 4d, with the corresponding AC impedance plots displayed in the inset. The cell exhibited an initial current of 25 μA that stabilized at 15 μA, while the fitted R_ct_ before and after polarization were 380 Ω and 548 Ω, respectively. The calculated t_Li+_ value for 1-0.3PTHF was 0.19, determined using the Bruce–Vincent–Evans equation. In addition, the rate capability performance was evaluated using cycling study of a Li symmetrical cell assembled with the in situ 1-0.3PTHF. The corresponding GCD profile under various current densities is reported in Figure 4e. The cell exhibited a negligible increment in polarization voltage plateau with rising current densities, without any sudden voltage drop until 3 mA cm^−2^, indicating the absence of internal short-circuits or dendrite-induced failure beyond critical current density (CCD) [12,18,30]. Yang et al. reported that a LiTa_2_PO_8_-based hybrid solid electrolyte exhibited a low CCD (~1.6 mA cm^−2^), which increased to 2 mA cm^−2^ upon introducing a small amount of liquid electrolyte to enhance Li–polymer interfacial contact [53], highlighting the importance of interfacial wettability and stability in suppressing dendrite propagation [18]. The in situ 1-0.3PTHF provides both interfacial wettability and stability at the Li–polymer interface, enabling stable cycling at current densities up to 3 mA cm^−2^ without interruption and demonstrating the superior short-circuit resistance of the in situ formed GPE technique for practical quasi-solid-state LMBs.

### 3.3. LiClO_4_-Derived Interphase Formation for Uniform Li Deposition

**Figure 5:**
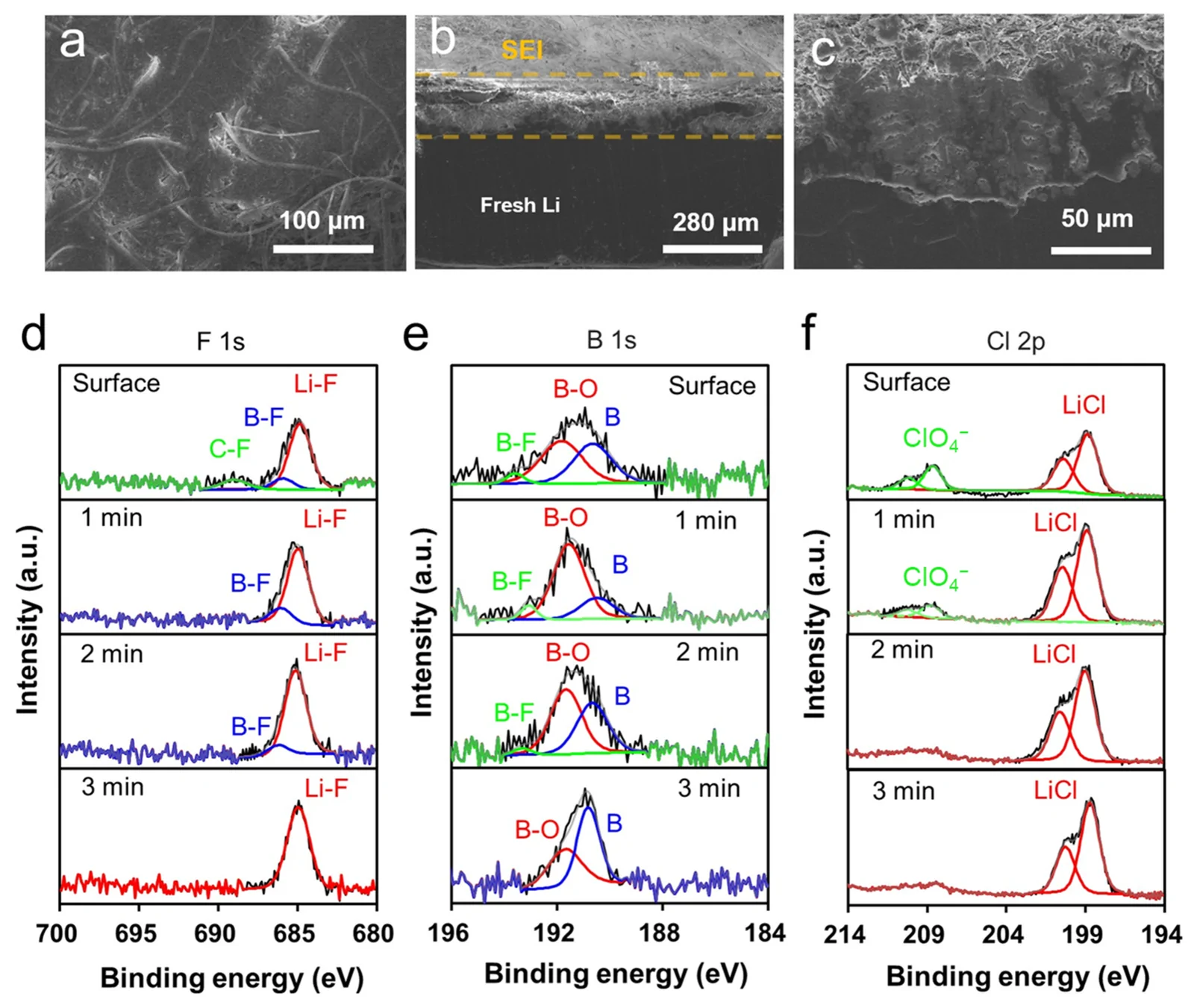
SEM images of harvested Li metal electrode after cycling: (**a**) top-view SEM and (**b**,**c**) cross-sectional SEM. In-depth XPS analysis of the harvested Li metal anode from the cycled Li symmetric cell using in situ 1-0.3PTHF, with etching time increased from 0 (surface) to 3 min: (**d**) F 1s spectra, (**e**) B 1s spectra, and (**f**) Cl 2p spectra.

When the current density was switched back to 2 mA cm^−2^, the overpotential remained nearly unchanged with negligible voltage relaxation between 60 h and 100 h, likely attributable to irreversible Li deposition and continuous SEI growth [18] contributing to additional interfacial resistance. To further probe interfacial reversibility, the cell was subjected to a recovery test at 0.1 mA cm^−2^ after the high-current-density cycling (Figure 4f). A significant decrease in overpotential was observed, followed by a slow gradual increase during extended cycling (over 190 h). Zhang et al. reported that imidazolium-based ionic liquid additives in PEO/LLZO composite SSEs promoted enhanced SEI formation and increased interfacial resistance under high current densities, which manifested as a sustained voltage plateau during reverse cycling [54]. The Li deposition behavior was further confirmed by interface analysis of the harvested Li surface. Surface SEM revealed non-mossy Li electrodeposits (Figure 5a), while EDS analysis detected predominant carbon, fluorine, boron, and chlorine signals (Figure S6), consistent with SEI-derived products. Cross-sectional SEM (Figure 5b) further showed that the SEI layer was primarily confined to the surface, and the bulk Li retained its structural integrity. The high-magnification cross-sectional SEM (Figure 5c) further revealed a dense and compact SEI layer.

In-depth XPS analysis was employed to further characterize the chemical composition of SEI products. The aliphatic carbon (C-C) in the C 1s spectrum was referenced to 284.8 eV for binding energy calibration. Figure 5d–f depict the XPS spectra of F 1s, B 1s, and Cl 2p collected after Ar^+^ sputtering for 0 min (surface), 1 min, 2 min, and 3 min, respectively. The XPS spectra of F 1s (Figure 5d) exhibited three resolved peaks at 684.8, 685.9, and 689 eV corresponding to LiF, B-F, and C-F units, respectively. The Li-F unit originated from the decomposition of BF_3_ species, whereas the B-F unit was assigned to residual, unreacted BF_3_ [32]. The C-F species was inferred to be a coordinated compound between BF_3_ species and organic segments rather than a SEI product, as its signal was detected only at the outermost surface. In addition, the B 1s spectra (Figure 5e) was dominated by B-O (191.8 eV) and B complexes (190.6 eV), which were generated from decomposition of BF_3_. These boron-containing species have been reported to stabilize the Li metal interface [31,32]. The Cl 2p spectra exhibited two doublets. Based on the reported binding energies [55], the lower binding energy was assigned primarily to LiCl unit or other reduced inorganic chloride species, whereas the higher binding energy represents ClO_4_^−^ anion, as shown in Figure 5f. The ClO_4_^−^ anion and LiCl were ascribed to precipitation and decomposition of LiClO_4_ [55]. Previous studies have reported that LiCl exhibits a lower Li^+^ diffusion barrier than LiF and a wide ESW, suggesting that LiCl-containing interphases may facilitate Li^+^ transport and contribute to more uniform Li deposition [56,57]. In the present study, however, XPS confirms only the presence of LiCl and does not directly determine its contribution to interfacial Li^+^ transport.

The signal of ClO_4_^−^ anion decreased with increasing etching time, indicating precipitation of unreacted salt was distributed close to the outer surface. Consistently, SEI species remained dominant throughout the depth profile, and just minor signals of residual unreacted species were detected at the outer surface across F 1s, B 1s, and Cl 2p spectra. Morphological and compositional analyses collectively indicate the formation of a dense and protective SEI film enriched with boron-containing and LiCl components. Specifically, SEM supports a compact morphology, whereas XPS depth profiling confirms a chemically homogeneous interphase with minimal residual precursor species. The coexistence of boron-containing species and LiCl in the SEI may help mitigate the polarization associated with LiF-rich SEIs alone [56]. As discussed above, previous studies have associated boron-containing species with stabilization of the Li interface and LiCl with favorable Li^+^ transport. Together, these findings provide a plausible explanation for the outstanding interfacial stability of the in situ 1-0.3PTHF electrolyte during prolonged cycling at high current densities.

**Figure 6:**
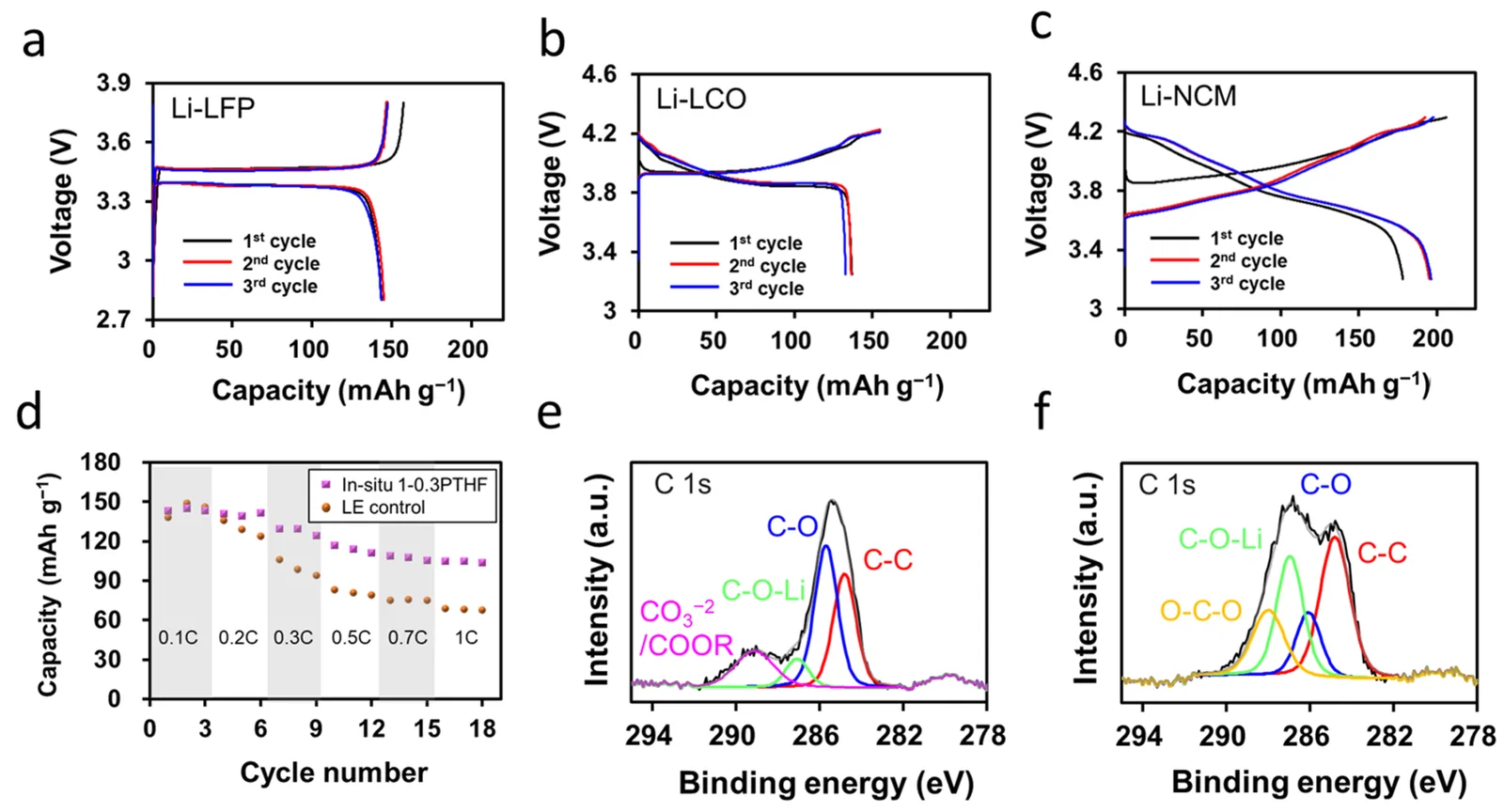
Galvanostatic charge–discharge curves at 0.1C for (**a**) Li-LFP, (**b**) Li-LCO, and (**c**) Li-NCM cells using the GF-supported in situ 1-0.3PTHF GPE. (**d**) Rate performance of the Li-LFP cell with GF-supported in situ 1-0.3PTHF GPE and LE control. XPS analysis of the harvested Li metal anode from the cycled Li-LFP cell using (**e**) the LE control electrolyte and (**f**) the in situ 1-0.3PTHF GPE.

Given the electrochemical performance of the in situ 1-0.3PTHF GPE, its feasibility in Li-metal full cells was further evaluated using LiFePO_4_ (LFP), LiCoO_2_ (LCO), and LiNi_0.8_Co_0.1_Mn_0.1_O_2_ (NCM) cathodes. In Figure 6a–c, the initial galvanostatic charge–discharge profiles of the Li/1-0.3PTHF/LFP, Li/1-0.3PTHF/LCO, and Li/1-0.3PTHF/NCM cells at 0.1C demonstrated the preliminary feasibility of the in situ 1-0.3PTHF GPE employing practical cathode chemistries.

The rate performance of Li-LFP cells assembled with in situ 1-0.3PTHF was further compared with that of an unpolymerized liquid-electrolyte (LE) control consisting of 1 M LiClO_4_ in THF without BF_3_-THF. The cells were evaluated at rates from 0.1C to 1C between 3.0 and 3.8 V, as shown in Figure 6d. The in situ 1-0.3PTHF cell delivered average discharge capacities of 144.0, 141.6, 127.8, 114.0, 107.5, and 104.6 mAh g^−1^ at 0.1C, 0.2C, 0.3C, 0.5C, 0.7C, and 1C, respectively, whereas the LE control delivered 147.6, 129.7, 99.7, 81.1, 75.4, and 68.2 mAh g^−1^. Accordingly, the in situ 1-0.3PTHF cell maintained 72.6% of its discharge capacity from 0.1C to 1C, compared with 46.2% for the LE control. The greater capacity loss of the LE control may be associated with interfacial decomposition of liquid THF and the resulting corrosion of the Li anode (Figure S7a). In the C 1s spectrum of the Li anode cycled with the LE control (Figure 6e), the component at 289.1 eV was assigned to CO_3_^2−^ and COOR species, consistent with the formation of oxidized carbonaceous products. In contrast, the anode cycled with in situ 1-0.3PTHF reflected metallic integrity (Figure S7b) and no pronounced component near 289 eV. Instead, the C 1s spectrum was dominated by PTHF-related carbon species (Figure 6f). These XPS results suggested reduced THF-derived interfacial decomposition following in situ PTHF formation, contributing to a more stable Li–electrolyte interface under the tested rate conditions.

## 4. Conclusions

In this work, we report a two-component system for fabricating in situ polymerized 1-0.3PTHF, in which SEI-favorable BF_3_-THF initiator is employed at a markedly reduced concentration (0.3 M), and 1 M LiClO_4_ serves as both the Li^+^ source and polymerization-assisting additive. It was found that LiClO_4_ impacted on the structure of the synthesized PTHF, probably through ionic coordination between LiClO_4_ and growing PTHF chains, leading to a narrow molecular weight distribution (Đ = 1.23) and small crystalline domains among PTHF segments compared to the commercial PTHF (Đ = 3.25). The introduction of LiClO_4_ enhanced ionic conductivity by approximately an order of magnitude (10^−3^ S cm^−1^), while CV of in situ 1-0.3PTHF exhibited a sloped cathodic current and increased anodic peak current density (from 10 to 215 mA cm^−2^) compared to those of bare 0-2.232PTHF. The Li symmetric cell using in situ 1-0.3PTHF was capable of cycling stepwise up to 3 mA cm^−2^ without interruption, which is associated with excellent interfacial contact and inorganic-rich SEI protection. Post-mortem SEM and XPS analyses further revealed uniform Li deposition together with the formation of a dense SEI layer enriched with B–O, B complex, and LiCl species. Full cell evaluations with LFP, LCO, and NCM cathodes demonstrated the preliminary feasibility of 1-0.3PTHF across different cathode chemistries. This work provides new insights into the multifunctional role of LiClO_4_ beyond that of a lithium-ion source during in situ polymerization. The resulting 1-0.3PTHF electrolyte integrates polymerization regulation, enhanced ionic conduction, and protective SEI formation, offering a new perspective on the role of lithium salts in in situ polymerized GPEs.

## Data Availability

The original contributions presented in this study are included in the article/Supplementary Materials. Further inquiries can be directed to the corresponding author.

## Supplementary Materials

The following supporting information can be downloaded at https://www.mdpi.com/article/10.3390/polym18192379/s1.
