# Supplementary material for: Multifunctional LiClO4 for Polymerization Regulation, Ionic Conduction, and Interphase Formation in In Situ Poly(tetrahydrofuran) Gel Polymer Electrolytes
Source: Polymers (Basel). 2026 Sep 29;18(19):2379. doi: 10.3390/polym18192379 (PMC13666992; doi:10.3390/polym18192379)
Supplement: Supplementary file 1 [file polymers-18-02379-s001.zip › polymers-4560457-supplementary.pdf]

Supplemental Information

Multifunctional $LiClO_4$ for polymerization regulation, ionic conduction, and interphase formation in in-situ poly(tetrahydrofuran) gel polymer electrolytes

1. Materials

THF (anhydrous, ≥99.9%, inhibitor-free), BF_3-THF, and PTHF (with an average M_n of 2,900), Bis(trifluoromethane)sulfonimide lithium salt (LiTFSI, 99.95% trace metals basis) were purchased from Sigma-Aldrich. Lithium perchlorate ($LiClO_4$, anhydrous, ACS, 95% min) was purchased from Alfa Aesar (Ward Hill, MA, U.S). Li metal anode (>99.95%, 450 µm in thickness, 15.8 mm diameter) was purchased from UBIQ (Taoyuan City, Taiwan). The LFP composite cathode with active LFP mass loading approximately 3.0 mg cm^{-2} was obtained from Battery Research Center of Green Energy (Ming Chi University of Technology, New Taipei City, Taiwan). Commercial cathode electrodes (NCM811 and LCO foil) were purchased from UBIQ (Taoyuan City, Taiwan). The typical active material mass loadings for NCM811 and LCO were 11.8 and 12.5 mg cm^{-2}, respectively.

2. Characterization

X-ray diffraction (XRD) was performed on a Bruker D2 Phaser (Bruker AXS GmbH, Karlsruhe, Germany) with Cu Kα radiation (λ = 1.54 Å) over the 2θ range of 10–80°. The surface morphology was examined by field-emission scanning electron microscopy, and elemental analysis was conducted using energy-dispersive X-ray spectroscopy (EDS) (FE-SEM, SU8000, Hitachi Ltd., Tokyo, Japan). The oscillatory shear tests were measured using a TA Instruments (New Castle, DE, USA) DHR-2 rheometer equipped with an 8 mm parallel plate. Frequency sweep tests were conducted at room temperature over an angular frequency range of 0.1-100 rad s^{-1} with a fixed strain amplitude of 5% at room temperature. Differential scanning calorimetry (DSC) was analyzed using Q20 (TA Instrument, New Castle, DE, USA) with approximately 5 mg of sample, heated at 10 °C min^{-1} under a nitrogen purge (50 mL min^{-1}). Nuclear Magnetic Resonance (NMR) was carried out on DD2 600 MHz NMR Spectrometer (Agilent, Santa Clara, CA, USA) using $CDCl_3$ as the solvent. Gel-permeation chromatograph (GPC) was tested on Nexara LC-40D XR (Shimazu, Kyoto, Japan) using THF as the mobile phase.

3. Electrochemical Measurements

Electrochemical impedance spectroscopy (EIS) was performed using an alternating

current (AC) method using Autolab (PGSTAT-30 Eco Chemie B. V., Utrecht, Netherlands) with a voltage amplitude of 10 mV over a frequency range of 0.1 Hz to 0.1 MHz at RT. The ionic conductivity of the PTHF-based GPEs was determined from the bulk resistance obtained from the Nyquist plot. The electrochemical stability window (ESW) and cyclic voltammetry (CV) measurements of the glass fiber (GF)-supported 1-0.3PTHF GPE were also carried out at room temperature using the same instrument, employing a stainless-steel (SS) working electrode (15.5 mm in diameter) and lithium foil as both counter and reference electrodes. The ESW was recorded at a scan rate of 1 mV s^{-1} over a potential range of 3.0–7.0 V versus Li/Li^+, while CV was performed at a scan rate of 10 mV s^{-1} from −1.0 to 1.0 V versus Li/Li^+. The lithium-ion transference number (t_{Li^+}) of the PTHF membrane was evaluated using a symmetric Li/Li cell under potentiostatic polarization at a small applied potential of 0.01 V at RT. Galvanostatic charge/discharge (GCD) test was performed on symmetric Li/Li coin cells (CR2032 type) using a Model BAT-750B (AcuTech System Co. Ltd., New Taipei City, Taiwan) at RT. The cell was cycled under stepwise current densities ranging from 2.0 to 3.0 mA cm^{-2}, with each step maintained for 0.5 h, and then the current densities were reversed sequentially back to 2.0 mA cm^{-2}. Subsequently, the cell was cycled at 0.1 mA cm^{-2} to evaluate the recovery behavior after high-current-density cycling. After the unit cell (Li-LFP battery, Li-LCO battery, and Li-NCM battery) was assembled in a CR2032-type coin cell in an argon gas-filled glove box, they were stored for 48 h before measuring electrochemical performance. Galvanostatic charge–discharge tests were conducted at room temperature using a BAT-750B battery tester. Three initial cycles were performed at 0.1C over voltage ranges of 2.8-3.8 V for Li-LFP, 3.25-4.3 V for Li-LCO, and 3.2-4.3 V for Li-NCM cell. The rate capability of the Li-LFP cell was subsequently evaluated at 0.1, 0.2, 0.3, 0.5, 0.7, and 1C between 3.0 and 3.8 V. GF-supported 1 M $LiClO_4$ in THF without BF_3-THF was used as the LE control.

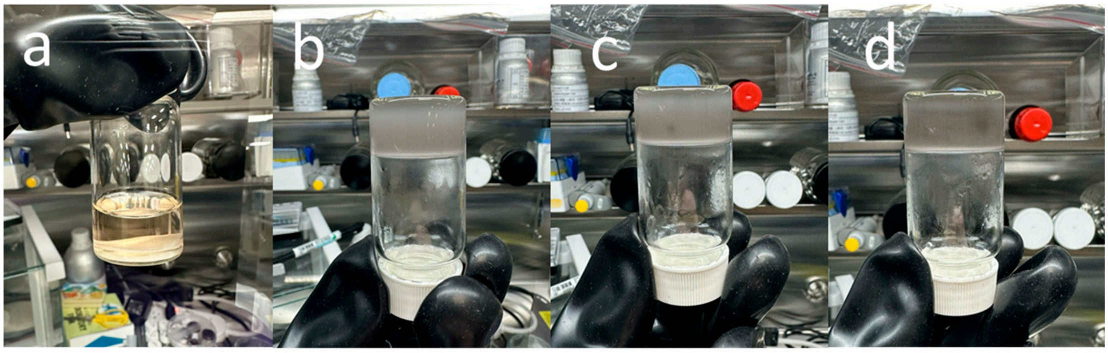

Figure S1. Digital images of (a) polymeric precursor and steady-state LiTFSI–PTHF gels prepared with 2.232 M BF_3–THF initiator and LiTFSI additions of (b) 0.2 M, (c) 0.25 M, and (d) 0.3 M. Inspired by the electrolyte-salt-induced cationic ring-opening polymerization, we conducted a preliminary study by incorporating small amounts of LiTFSI into bare PTHF containing 2.232 M BF_3-THF (0-2.232PTHF). This leverages the Lewis acidity of lithium salts to activate ring-opening polymerization reaction. Figure S1 shows digital images of the obtained PTHF consisting of 2.232 M BF_3-THF with the addition of 0.2 M, 0.25 M, and 0.3 M LiTFSI. Encouragingly, the polymeric precursors were of high viscosity after 3 h, and transformed into solid-like gel after 6 h from initiation. Clearly, the bulk transformation was accelerated in the presence of the Li-conducting salt. The success of LiTFSI as a catalytic promoter in THF polymerization is one of the tests that support our hypothesis using the combination of a catalytic Li salt and the functional BF_3-THF initiator to initiate ring-opening polymerization of THF.

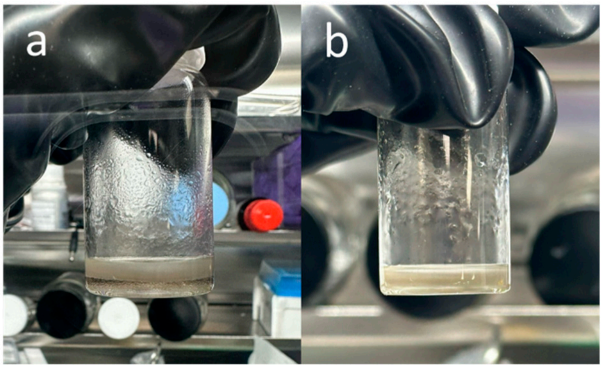

Figure S2. Digital images of steady-state (a) LiTFSI–PTHF gel and (b) $LiClO_4$–PTHF gel, both composed of 1 M salt (LiTFSI or $LiClO_4$) and 0.6 M BF_3–THF. Preliminary studies revealed that the LiTFSI–PTHF system could not form a uniform gel at a typical salt concentration (1 M LiTFSI). Instead, localized viscosity variations were observed during gelation, despite the reduced BF_3–THF content. Supporting evidence was obtained from a LiTFSI-PTHF system containing 1 M LiTFSI and 0.6 M BF_3-THF, as shown in Figure S2a. The resulting gel appeared as a separate phase at the top of polymeric solution, while the remaining portion remained fluid. This result indicated heterogeneous and incomplete THF polymerization, suggesting a synthesis limitation at certain LiTFSI-to-BF_3-THF ratio. In contrast, the $LiClO_4$-PTHF system under the same conditions (1 M $LiClO_4$ and 0.6 M BF_3-THF) produced a uniform PTHF gel at the steady state (Figure S2b), although transient viscosity difference was observed during the early stage of polymerization (as indicated in Figure 1a). Overall, $LiClO_4$ salt acted as a more effective co-initiating agent, yielding a uniform PTHF product across a wide range of salt to BF_3-THF compositions.

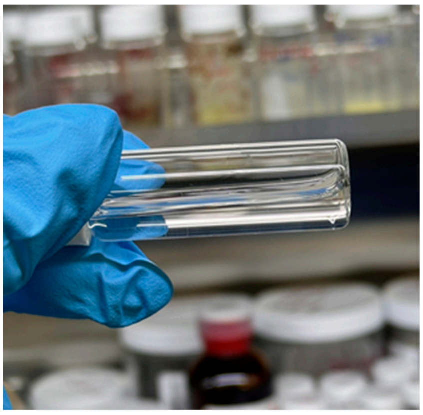

Figure S3. Digital images of 0.3 M BF_3-THF in THF (0-0.3PTHF) after 48 h from the preparation of polymeric solution (without $LiClO_4$).

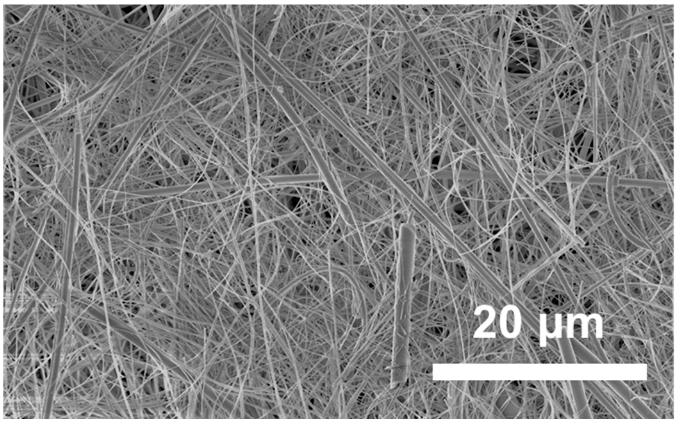

Figure S4. SEM image of pristine glass fiber (GF) support.

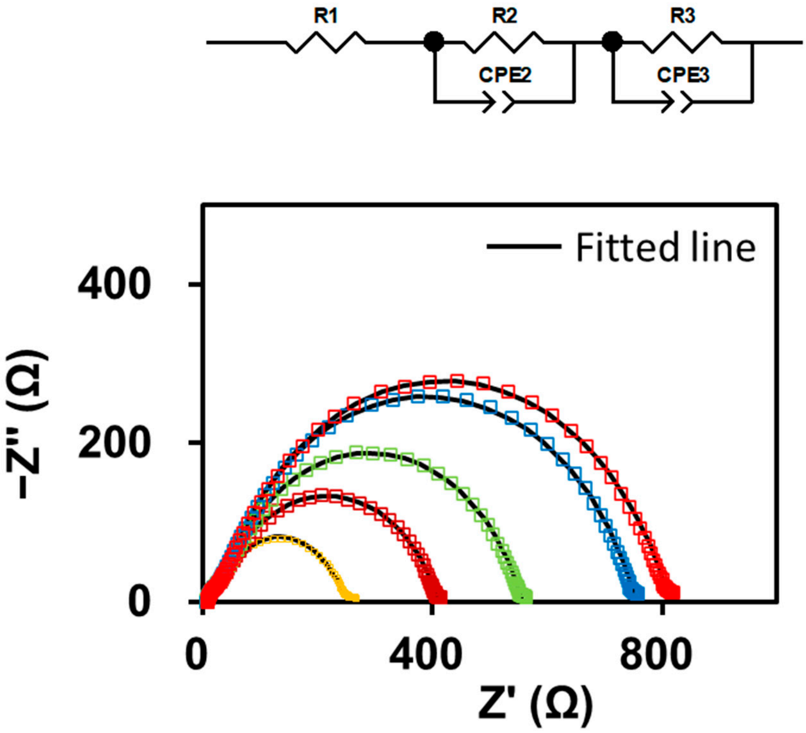

Figure S5. Equivalent circuit model used for fitting the measured Nyquist plots of lithium symmetrical cell. The fitted bulk resistance (R_b) corresponds to R1, while the charge-transfer resistance (R_{ct}) is obtained from the combined contributions of R2 and R3 in the model. The experimental and fitted Nyquist plots of the Li symmetric cell using 1-0.3PTHF electrolyte after aging for 8, 12, 24, 36, and 48 h, shown from left to right.

a

b

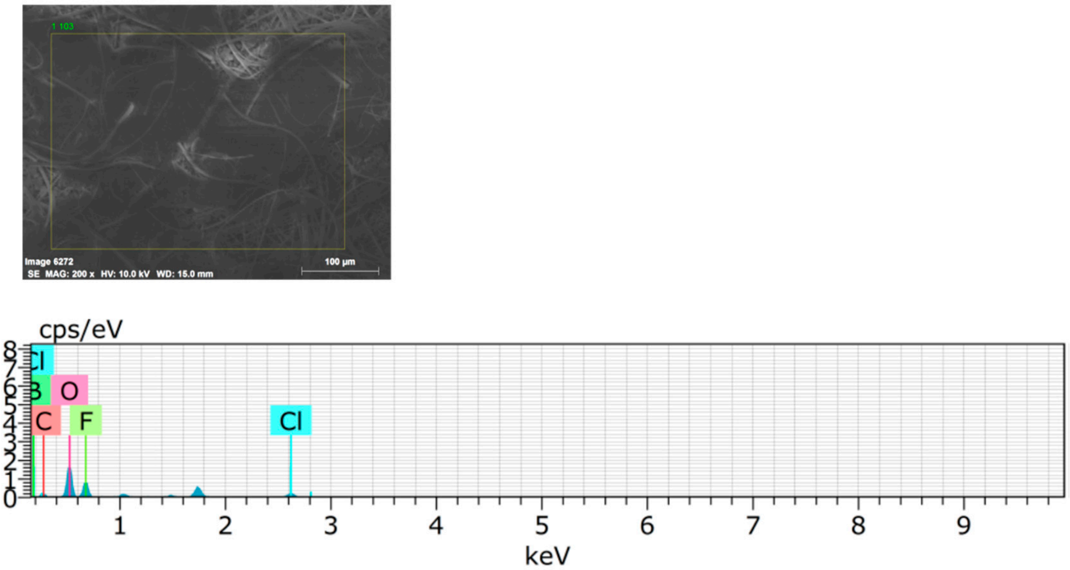

Figure S6. SEM image and EDS analysis of harvested Li metal electrode from the cycled Li symmetrical cell.

a b

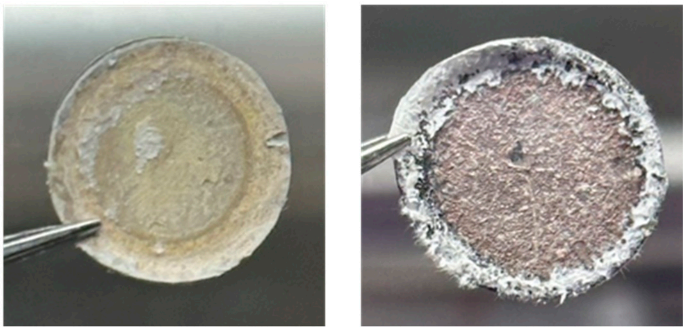

Figure S7. SEM images of the harvested Li metal from cycled Li-LFP cells using: (a) LE control, and (b) in-situ 1-0.3PTHF as electrolyte.
